# Complete Genome Sequence of Crohn's Disease-Associated Adherent-Invasive *E. coli* Strain LF82

**DOI:** 10.1371/journal.pone.0012714

**Published:** 2010-09-17

**Authors:** Sylvie Miquel, Eric Peyretaillade, Laurent Claret, Amélie de Vallée, Carole Dossat, Benoit Vacherie, El Hajji Zineb, Beatrice Segurens, Valerie Barbe, Pierre Sauvanet, Christel Neut, Jean-Frédéric Colombel, Claudine Medigue, Francisco J. M. Mojica, Pierre Peyret, Richard Bonnet, Arlette Darfeuille-Michaud

**Affiliations:** 1 Clermont Université, Université d'Auvergne, JE2526, INRA, USC-2018, Clermont-Ferrand, France; 2 Institut Universitaire de Technologie, Université d'Auvergne, Aubière, France; 3 Laboratoire: Microorganismes Génome et Environnement, Université Clermont 2, CNRS, UMR 6023, Aubière, France; 4 Commissariat à l'Energie Atomique (CEA), Direction des Sciences du Vivant, Institut de Génomique, Genoscope, Evry, France; 5 Centre Hospitalier Universitaire, Pôle digestif, Clermont-Ferrand, France; 6 INSERM U995, Lille, France; 7 CNRS-UMR 8030, Laboratoire d'Analyse Bioinformatique en Génomique et Métabolisme, Evry, France; 8 Departamento de Fisiología, Genética y Microbiología, Universidad de Alicante, Alicante, Spain; 9 Centre Hospitalier Universitaire, Bactériologie, Clermont-Ferrand, France; University of Hyderabad, India

## Abstract

**Background:**

Ileal lesions of Crohn's disease (CD) patients are abnormally colonized by pathogenic adherent-invasive *Escherichia coli* (AIEC) able to invade and to replicate within intestinal epithelial cells and macrophages.

**Principal Findings:**

We report here the complete genome sequence of *E. coli* LF82, the reference strain of adherent-invasive *E. coli* associated with ileal Crohn's disease. The LF82 genome of 4,881,487 bp total size contains a circular chromosome with a size of 4,773,108 bp and a plasmid of 108,379 bp. The analysis of predicted coding sequences (CDSs) within the LF82 flexible genome indicated that this genome is close to the avian pathogenic strain APEC_01, meningitis-associated strain S88 and urinary-isolated strain UTI89 with regards to flexible genome and single nucleotide polymorphisms in various virulence factors. Interestingly, we observed that strains LF82 and UTI89 adhered at a similar level to Intestine-407 cells and that like LF82, APEC_01 and UTI89 were highly invasive. However, A1EC strain LF82 had an intermediate killer phenotype compared to APEC-01 and UTI89 and the LF82 genome does not harbour most of specific virulence genes from ExPEC. LF82 genome has evolved from those of ExPEC B2 strains by the acquisition of *Salmonella* and *Yersinia* isolated or clustered genes or CDSs located on pLF82 plasmid and at various loci on the chromosome.

**Conclusion:**

LF82 genome analysis indicated that a number of genes, gene clusters and pathoadaptative mutations which have been acquired may play a role in virulence of AIEC strain LF82.

## Introduction

Crohn's disease (CD) is a chronic inflammatory bowel disease in humans which has features that might be the result of a microbial process in the gut [1], [2], [3], [4]. Various studies have addressed the hypothesis that pathogenic bacteria contribute to the pathogenesis of inflammatory bowel disease [4], [5], [6], [7], [8]. *Escherichia coli* strains have been assigned a putative role in the pathogenesis of CD. Increased numbers of mucosa-associated *E. coli* forming a biofilm on the surface of the gut mucosa, are observed in patients with CD [9], [10], [11], [12], [13], [14], [15]. Most of the *E. coli* strains colonizing the intestinal mucosa in patients with inflammatory bowel disease belong to the B2 and D phylogroup [11] and strongly adhere to intestinal epithelial cells [10], [12]. In addition, seven independent studies have reported the presence of intramucosal *E. coli* or mucosa-associated *E. coli* with invasive properties in CD patients [12], [16], [17], [18], [19], [20], [21]. On the basis of the pathogenic traits of CD-associated *E. coli*, a pathogenic group of *E. coli* was designated AIEC for Adherent-Invasive *Escherichia coli* [22]. The criteria for inclusion in the group are: (i) ability to adhere to and to invade intestinal epithelial cells with a macropinocytosis-like process of entry dependent on actin microfilaments and microtubule recruitment, (ii) ability to survive and to replicate extensively in large vacuoles within macrophages without triggering host cell death, and (iii) ability to induce the release of large amounts of TNF-α by infected macrophages. The high level of ileal colonization in CD patients by AIEC is linked to the abnormal expression of the glycoprotein CEACAM6 which acts as a receptor for AIEC adhesion via type 1 pili [23], [24].

The prototype strain for AIEC pathovar is *E. coli* strain LF82. This reference AIEC strain is included in most, if not all, of the studies analysing of *E. coli* strains associated with Crohn's disease performed by our group [25], [26] or others [12], [16], [17], [27], [28], [29], [30], [31], [32], [33]. This, combined with the virulence properties of AIEC strain LF82 [22], [24], [34], [35], led us to decipher the genome sequence of AIEC reference strain LF82 to compare it with the other known *E. coli* genome sequences and with as other bacteria of the *Enterobacteriaceae* family having an intracellular lifestyle in eukaryotic cells.

## Results and Discussion

### Overview of AIEC strain LF82 genome

**Figure 1:**
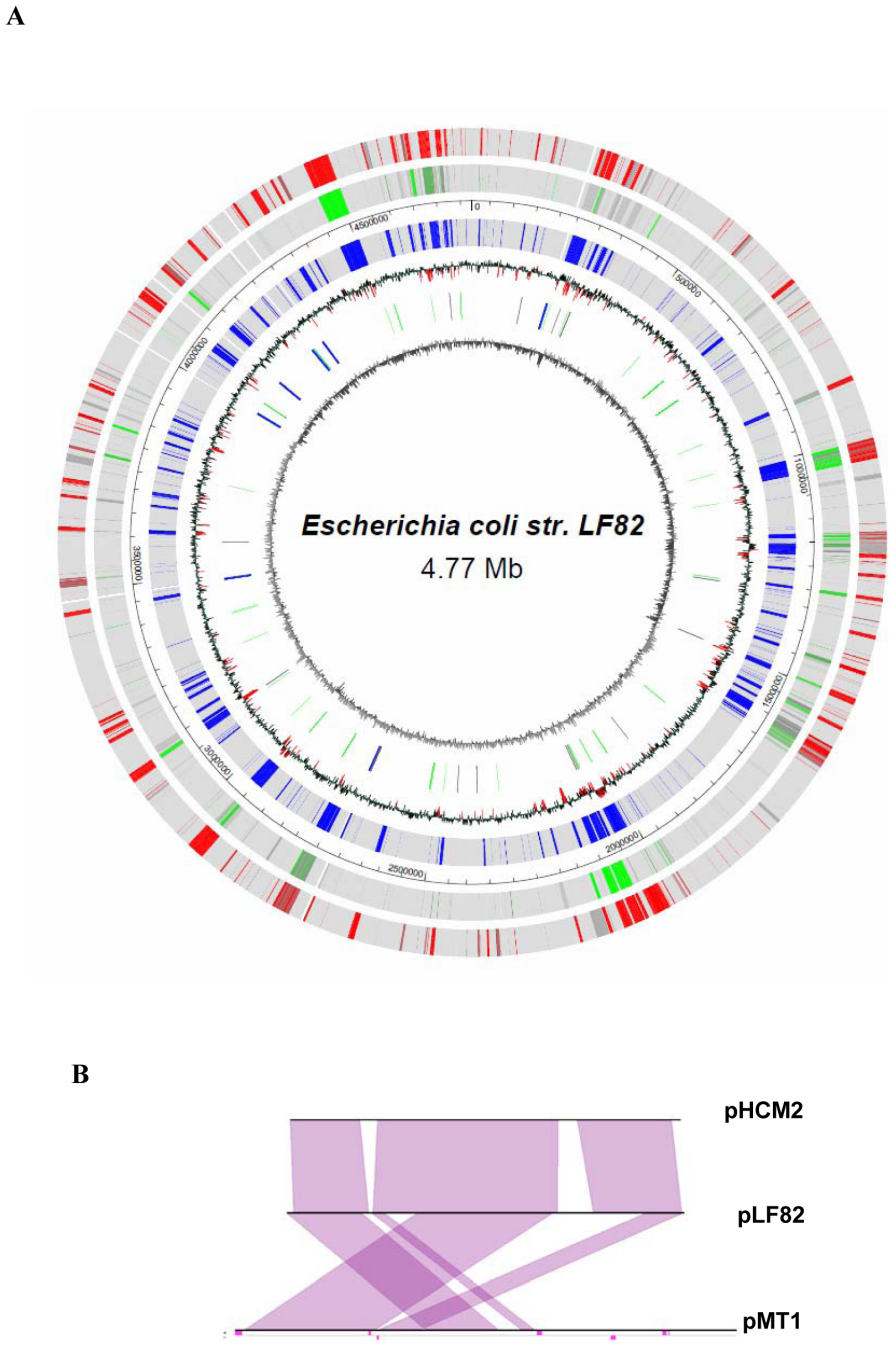
AIEC strain LF82 chromosome circular map and synteny plots between pLF82 and pHCM2 and pMT1. Circular representation of the *E. coli* LF82 genome (A). Circles display from the inside out: (1) GC skew (G+C/G−C using a 1 kbp sliding window). (2) Location of tRNAs (green), rRNAs (blue) and Insertion Sequences (grey). (3) GC deviation (mean GC content in a 1 kbp window – overall mean GC). Red areas indicate that deviation is higher than 2 Standard Deviation. (4) (5) and (6) Gene specificity of LF82 strain at strain level (K12, in blue), and at group level: *E. coli* B2 strains, in green, and *E. coli* commensal strains, in red. Genes sharing at least one homolog in an other *E. coli* of the same group and having more than 85 percent identity on at least 80% of its length were regarded as non specific. Synteny plots between the *E. coli* LF82 plasmid and the plasmid from *Salmonella enterica* serovar Typhi (upper comparison) and the plasmid from *Yersinia pestis* Pestoides (bottom comparison) (B). Synteny groups containing a minimum of five genes are shown in purple for colinear regions.

The genome of AIEC strain LF82 of 4,881,487 bp total size contains a circular chromosome with a size of 4,773,108 bp and a plasmid of 108,379 bp (Figure 1A). It contains 4376 CDSs corresponding to 88.3% of the complete chromosome. The number of CDSs in LF82 is low compared to that of other pathogenic *E. coli* strains involved in urinary tract infection (UTI), diarrhea or meningitis in humans or colibacillosis in chickens and closer to that of pathogenic APEC strain (Table 1). The GC content of the LF82 chromosome, about 50%, is close to that of all other complete sequenced *E. coli* genomes. In contrast, the plasmid sequence has a lower GC% content of 46.1% (Table 2), indicating that it could have been acquired by horizontal transfer from a distant species. Annotation step identified 121 CDSs that were closely similar to CDSs located on pMT1 plasmid from *Yersinia* species [36] and pHCM2 plasmid from *Salmonella enterica serovar* Typhimurium [37] (Table S1). Comparative genomic analysis using MaGe software has revealed a high synteny rate between LF82 plasmid and pHCM2 (synton 71%). However, for pMT1 plasmid synteny were only observed for 50% of this plasmid sequence with sequence inversion (Figure 1B). In addition, although the GC% of plLF82 was lower than that of plasmids pMT1 or pHCM2, of the 121 CDS identified on plLF82, 97 (∼80%) and 65 (∼45%) were also found on pHCM2 and pMT1 plasmids, respectively. Of note, 24 CDSs were common to these three plasmids and were not found in any other available genome sequences.

**Table 1:** General features of the adherent-Invasive *E. coli* LF82 genome compared with those of other sequenced B2 or K-12 *E. coli* strains.

Strains	Phylogroup	Pathotype (Host)	Clinical condition	Chromosome size (bp)	DNA coding sequence %	No. of CDSs	G+C Content (%)	No. of rRNA genes	No. of tRNA genes	No. of misc. RNAs^a^	Chromosome	Plasmids	Plasmid	Reference
			(sample)								GenBank accession no.	size (kb)	GenBank accession no.	
**LF82**	**B2**	AIEC	Crohn's disease	4,773,108	88.3	4,376	49.9	22	84	52	CU651637	108	CU638872	This study
		(Human)	(ileum)											
**536**		UPEC	Pyelonephritis	4,938,920	87.8	4,685	50.5	22	81	45	CP000247	-		[96]
		(Human)	(urine)											
**UTI89**		UPEC	Cystitis	5,065,741	91.1	5,066	50.6	22	88	ND^b^	CP000243	114	CP000244	[61]
		(Human)	(urine)											
**CFT073**		UPEC	Pyelonephritis	5,231,428	90.6	5,379	50.5	21	89	51	AE014075	-		[97]
		(Human)	(blood)											
**APEC_O1**		APEC	Colisepticemia	5,082,025	87.4	4,467	50.6	22	93	ND	CP000836	241	DQ517526	[59]
		(Chicken)	(lung)									174	DQ381420	
												103	CP000836	
												46		
**E2348/69**		EPEC	Children	4,965,553	85.5	4,703	50.8	22	92	73	FM180568	-	-	[98]
			(faeces)											
**ED1a**		-	Healthy subject	5,209,548	86.2	4,915	50.7	22	91	90	CU928162	119	CU928147	[48]
		(Human)	(faeces)											
**S88**		MNEC	New born meningitis	5,032,268	87.5	5,049	50.7	22	91	89	CU928161	134	CU928146	[48]
		(Human)	(cerebrospinal fluid)											
**MG1655**	**A**	K-12	Healthy subject	4,639,675	85.1	4,294	50.8	22	89	47	U00096	-		[99]
		(Human)	(faeces)											
**W3110**		K-12	Healthy subject	4,646,332	86.7	4,227	50.8	22	86	49	AP009048	-		Unpublished
		Human	(faeces)											

a Number of predicted miscellaneous RNAs.

b ND, not determined.

**Table 2:** Plasmid features of *E. coli* LF82 strain compared to highly conserved plasmids in *Salmonella enteritica* and *Yersinia pestis*.

	LF82	*Salmonella enteritica*	*Yersinia pestis*
**Plasmid names**	pLF82	phCM2	pMT1
**Genome size (bp)**	108379	106516	96210
**G+C content (%)**	46.17	50.61	50.23
**CDS number**	121	132	103
**Putative conserved domains detected**	52	ND	ND
**Hypothetical genes**	70	ND	ND
**Orphans**	8	ND	ND
**Orthologous genes to LF82**		97	55

### Phylogenetic position of strain LF82

**Figure 2:**
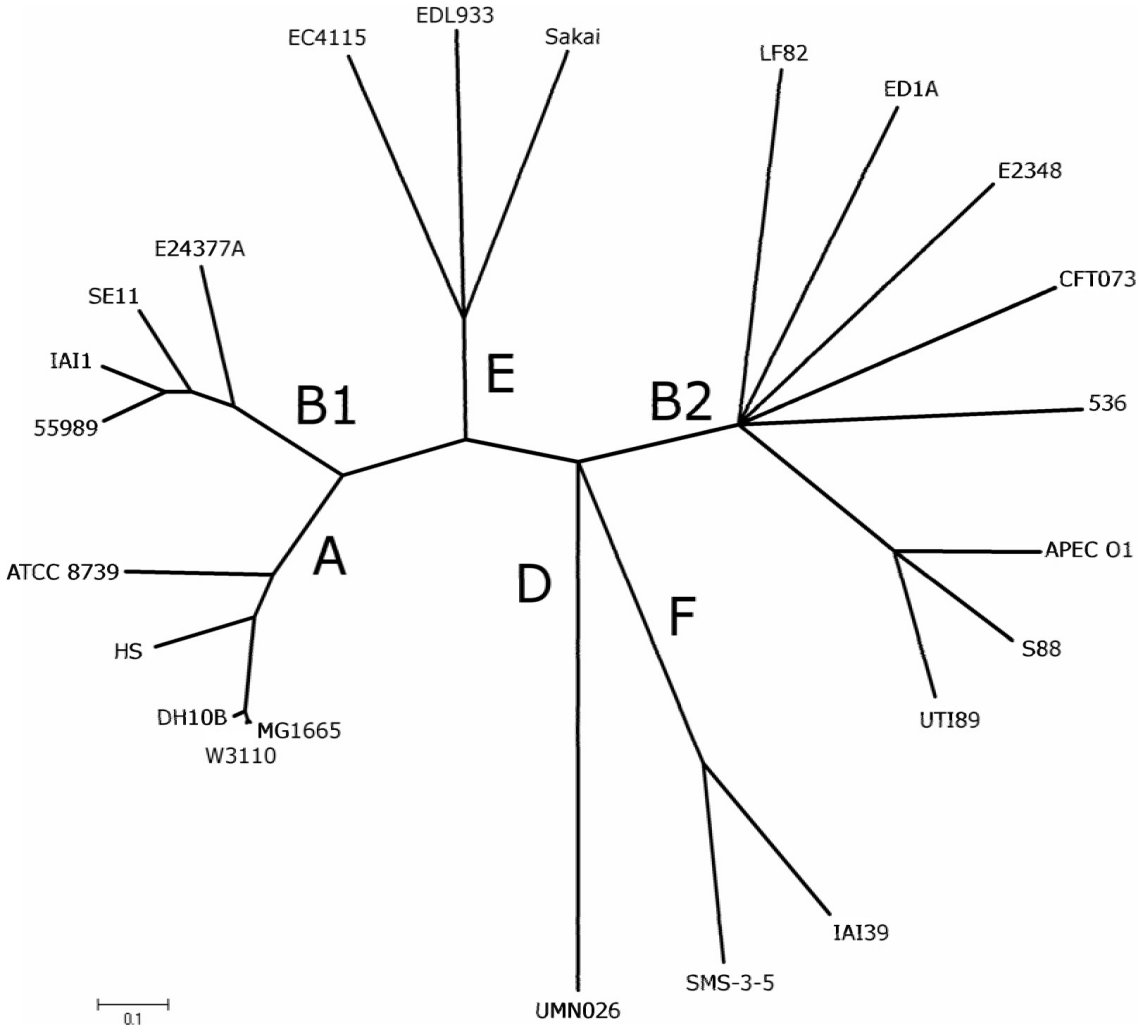
Recombination-insensitive phylogenetic analysis. The analysis was based on the sequence of seven house-keeping genes (7497 nucleotides from genes *arcA*, *aroE*, *icd*, *mdh*, *mtlD*, *pgi* and *rpoS*) of 23 genomes reference strains including LF82. The major branches are labeled according to the major phylogroups A, B1, B2, D, E and F.

*E. coli* strains are generally divided into four major phylogenetic groups A, B1, B2 and D [38], [39], although some strains may belong to additional groups [40], [41]. Recombination-insensitive phylogenetic analysis was undertaken with MLST data extracted from LF82 and 22 other *E. coli* genome sequences (Figure 2). The results confirmed the strong phylogenetic clustering of *E. coli* strains into six sharply separated branches, which could be equated to groups A, B1, B2, D, E and F [42]. Strain LF82 clustered with all the B2 extraintestinal pathogenic *E. coli* (ExPEC) strains involved in urinary tract infection, meningitis and avian colibacillosis. However, it formed a distinct clonal complex of this phylogenetic group B2, compared to B2 strains UTI89, S88 and APEC_O1, which clustered in a single subgroup.

**Figure 3:**
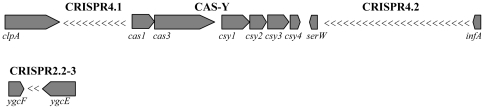
CRISPR regions of LF82 chromosome. Genes are shown as boxes pointing towards the direction of transcription. CRISPR repeats are represented by “<” symbols.

Clustered regularly interspaced short palindromic repeats (CRISPR) corresponding to redundant sequences that alternate with spacers of foreign origin [43], [44] were detected in LF82 chromosome (Figure 3). Two subtypes of CRISPR/CAS (CRISPR-associated genes) systems have been identified in *E. coli*, CRISPR2/CAS-E and CRISPR4/CAS-Y [45]. In LF82 we found a CRISPR4/CAS-Y system composed of a CRISPR4.1 array with 10 repeats and a CRISPR4.2 with 23 repeats, interspaced by a complete set of CAS-Y genes. In contrast, a CRISPR2.2-3 locus with two repeats was the only reminiscence of a CRISPR2/CAS-E system. Absence of both CRISPR2.1 and CAS-E genes is common to all strains of B2, although this is not a feature exclusive to the group. Among the *E. coli* reference collection ECOR; [46] and the available complete genomes analyzed here, CAS-Y genes have only been described in B2 strains (i.e. ECOR61, ECOR62, ECOR63, ECOR65, UTI89, APEC_01, S88 and ED1a) and B1 strain B7A. Identities to LF82 CRISPR4 spacers were found within the corresponding locus in ECOR61, ECOR62, UTI89, APEC_01, S88 (7 spacers each), ED1a (8 spacers) and ECOR65 (2 spacers).

### Global comparative genomic analysis

The organization of the LF82 genome is similar to that seen in other pathogenic *E. coli* strains with large regions of colinear *E. coli* core genome punctuated by genomic islands probably acquired by horizontal transfer. TBLASTN showed that 3132 CDSs (71.6%) out of the 4376 CDSs constitute the core genome of all *E. coli* strains used in this comparative genomic analysis (Table 3 and Table S2). This number is higher than that reported by Touchon *et al.* (1,996 genes) and Rasko *et al.* (2,200 genes) [47], [48]. This discrepancy is the consequence of the comparative approach used. We used TBLASTN analysis to compare predicted proteins of LF82 strain with the complete DNA sequence of the other strains. LF82 genomic comparative analysis also revealed the presence of conserved insertion-deletion inducing frameshifts in some core genes that may be used as new phylogroup markers (Table S3).

**Table 3:** Comparison of the “flexible genome” between AIEC LF82 strain and the other *E. coli* strains so for sequenced.

			LF82		
Phylogroup	Strain	Pathovar	Core genome	Number of CDS^a^	%^a^
B2	**536**	UPEC	3132 CDS (71.6%)	874	70.3
	**UTI89**			955	76.7
	**CFT073**			937	75.3
	**APEC_01**	APEC		968	77.8
	**E2348/69**	EPEC		714	57.4
	**ED1a**	commensal		791	63.6
	**S88**	MNEC		959	77.1
D	**UMN026**	ExPEC		656	52.7
	**IAI39**			630	50.6
	**SMS_3_5**	Environnemental		626	50.3
E	**EDL933**	EHEC		493	39.6
	**157_H7**			496	39.9
	**EC4115**			490	39.4
B1	**E24377A**	ETEC		522	42.0
	**55989**	EAEC		561	45.1
	**IAI1**	commensal		535	43.0
	**SE11**			540	43.4
ND	**ATCC 8739**			539	43.3
A	**HS**			496	39.9
	**DH10B**	K12		434	34.9
	**MG1655**			494	39.7
	**W3110**			493	39.6

a Number and percentage of CDSs within flexible genomes in common with AIEC strain LF82.

**Figure 4:**
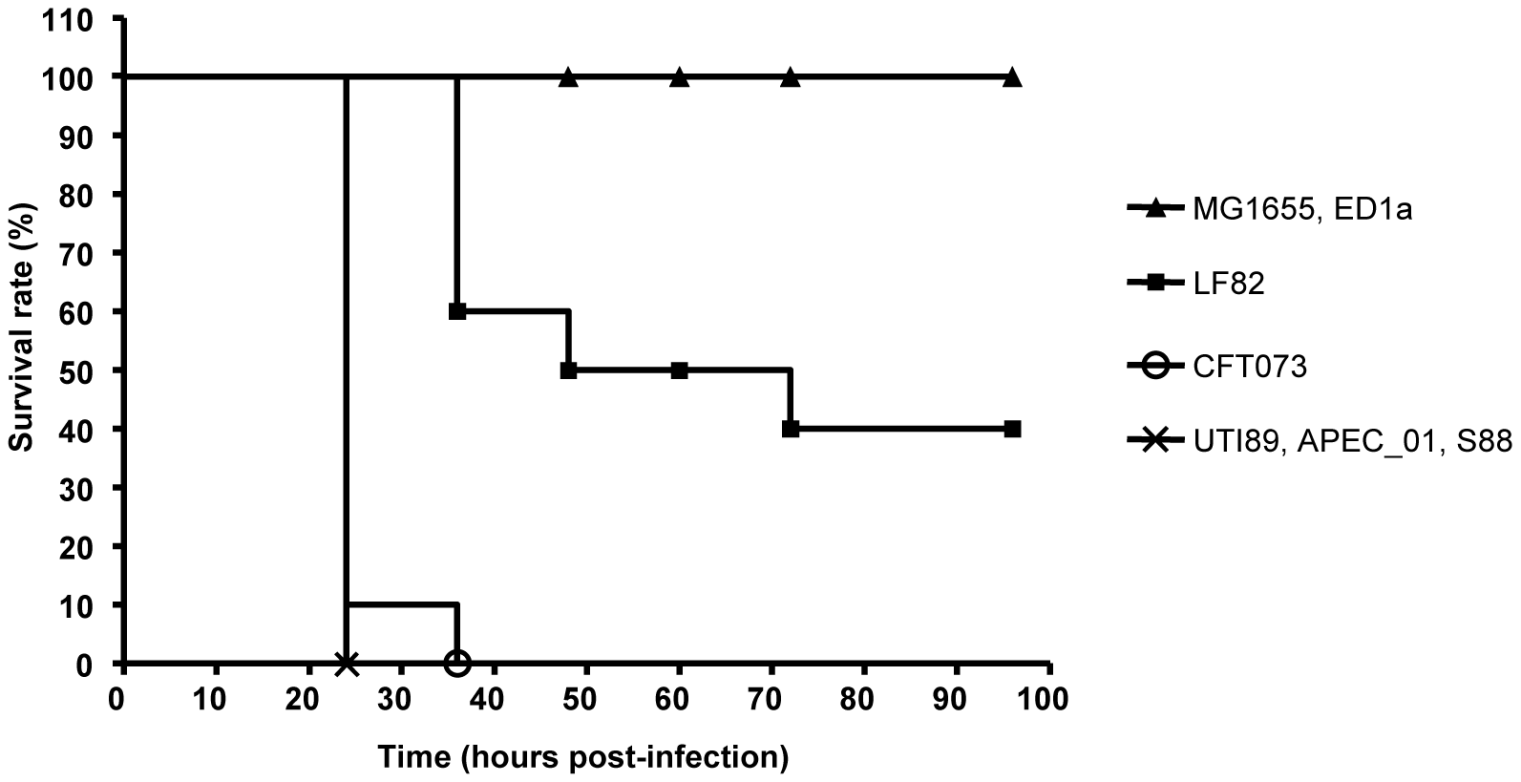
Evolution of survival rate of mice challenged subcutaneously with various *E. coli* strains. An inoculum of 2×10^8^ bacteria was injected in OF1 mice and 10 mice were used for each bacterial strain tested. Strains were classified as non-killer (<2 of 10 mice killed), killer (>8 mice killed) or intermediate.

Of the 1244 CDSs that are not encompassed in the core genome 1128 were found in at least one or more *E. coli* complete genomes and around 40% of them are present in commensal or K-12 *E. coli* strains. In addition, 33 CDSs (0.8%) were common to all B2 strains so far sequenced and formed four clusters of two to four genes in the genome (Table S2). Overall, the analysis of CDSs within the LF82 flexible genome indicated that this strain shared the highest percentages of common CDSs with strain APEC-01 isolated from lesions of chickens and turkeys clinically diagnosed with colibacillosis, followed by meningitis-associated *E. coli* strain S88 isolated from human cerebrospinal fluid, and by *E. coli* strain UTI89 isolated from human urine (Table 3). Of interest, strain UTI89, isolated from a patient with uncomplicated cystitis, was further assessed to be very close to meningitis-associated *E. coli* strains (Bingen, personnal communication). However, phenotype analysis in the mouse lethality model developed by Johnson *et al.* [49] indicated that A1EC strain LF82 had an intermediate killer phenotype compared to APEC-01, S88 and UTI89, which induced 100% lethality at 24h post-infection (Figure 4). Of note, in this model all the B2 strains were highly virulent except strains ED1a and EPEC strain E2348/69, which did not induce lethality.

We compared AIEC LF82 strain with other B2 *E. coli* strains and K-12 MG1655 strain using *RGPfinder* tool. The more interesting highly specific regions are summarized in Table 4 and ordered taking into account the specificity score found in the eight compared strains. Fifty-six LF82 CDSs are encompassed in one integrative element inserted near the *PheU* tRNA encoding gene and 14 in two very specific regions 1 and 2. These two regions showed no similarity with the compared *E. coli* strains with values of 100 meaning that these two LF82 regions are entirely specific. Interestingly, 115 AIEC LF82 CDSs (2.6%), not found in any *E. coli* genomes used in our comparative genomic analysis, were identified as LF82 unique CDSs (Table S4). In addition, 15 CDSs (0.3%) shared no homology with genes so far identified in any pathogenic bacteria. The presence of these CDSs, encoding mostly hypothetical proteins, indicated the high level of plasticity of the LF82 genome. It has now to be determined whether these CDSs allow AIEC bacteria to adapt to the human gut and/or are involved in generating chronic inflammation or whether the adaptation may be due to changes in gene expression of CDSs present in other bacterial strains.

**Table 4:** Regions of Genomic Plasticity of LF82 strain compared to B2 strains and K-12 MG1655 strain.

						B2							K12-MG1655	
						UPEC			APEC	EPEC	commensal	MNEC		
Region	Begin	End	Lenght (bp)	Features	Number of CDS (specifics CDS)	536	UTI89	CFT073	APEC_01	E2348/69	ED1a	S88		Description
				[Left] [Insinde] [Right]										
**1**	4128073	4133609	5537	[none] [none] [none]	6 (6)	100	100	100	100	100	100	100	100	Specific region 1
**2**	2122543	2128804	6262	[none] [none] [none]	8 (8)	100	100	100	100	88	88	100	100	Specific region 2
**3**	4454391	4503001	48611	[none] [int] [tRNA/int]	57 (56)	95	95	98	98	97	53	97	100	Integrative element
**4**	2047666	2079789	32224	[tRNA] [tRNA] [none]	26 (10)	82	73	79	85	79	76	79	76	Specific region 3
**5**	1296271	1303549	7279	[none] [tRNA] [tRNA]	8 (0)	89	100	22	100	22	89	100	78	Specific region 4
**6**	999027	1037235	38209	[none] [none] [none]	55 (8)	95	32	86	46	61	85	37	97	Prophage 1
**7**	1486962	1500101	13140	[pseudo] [none] [none]	14 (1)	41	35	29	35	100	53	35	71	Specific region 5
**8**	878063	888287	10225	[none] [none] [tRNA]	6 (0)	88	0	88	13	100	0	0	100	Specific region 6
**9**	2083448	2100892	17445	[none] [none] [none]	21 (0)	55	55	55	50	0	45	55	68	*pdu* operon
**10**	1535849	1552131	16283	[none] [none] [none]	16 (4)	50	28	33	33	50	67	39	61	fimbrial-like region
**11**	2735623	2769189	33567	[none] [none] [none]	45 (7)	100	50	21	42	17	19	10	92	Prophage 4
**12**	2925480	2960242	34763	[tRNA] [tRNA] [none]	26 (1)	19	15	11	19	81	81	19	93	PAI III
**13**	240866	275935	35070	[tRNA] [none] [IS]	29 (2)	10	10	87	10	100	20	10	87	PAI I

### Syntenic studies of integrative elements

Like other *E. coli* B2 genomes, LF82 genome has adopted the ‘mix and match’ evolution approach observed for UPEC [50]. As reported for most of the so far analyzed *E. coli* genomic islands, genomic islands of AIEC LF82 have a patchy structure, with the information segmented into modules that can be found independently in other locations of other genomes [47], [48]. Nine large genomic islands with a size larger than 17kb were identified in the LF82 genome, including the LF82 specific integrative element located at *PheU* tRNA (Table 5). Four of them were composed mainly of prophage-like elements, which being at the origin of the ongoing genetic diversity of many genomes could contribute to LF82 virulence [51].

**Table 5:** Comparison of integrative element features in the genome of LF82 strain with those of other sequenced B2 or K-12_MG1655 *E. coli* strains.

	Location						LF82	536	UTI89	CFT073	APEC_01	E2348/69	ED1a	S88	MG1655
Integrative elements	Start (pb)	Stop (pb)	tRNAs	Description	size (bp)	GC content (%)	Number of ORFs								
**Prophages**															
Prophage 1	999027	1036707	-		37681	47.3	54	2	25	4	26	16	6	23	1
Prophage 2	1173160	1232405	-		59246	48.3	80	69	56	68	59	32	45	59	31
Prophage 3	1582641	1632704	-		50064	48.3	54	11	37	32	44	31	11	41	22
Prophage 4	2736905	2769189	-		32285	51.3	45	0	0	32	0	33	38	36	1
**Putative pathogenicity islands**															
PAI I	240866	275935	*AspV*	T6SS	35070	50.1	29	26	26	2	26	0	23	26	3
PAI II	2007673	2041686	*AsnT*	Yersinia high pathogenicity island	34014	57.1	15	15	15	15	15	0	13	15	0
PAI III	2925480	2955960	*MetZ,V,W*	T6SS	30481	53.5	22	16	17	20	17	0	0	16	0
PAI IV	3110179	3128026	*PheV*	Group 2 capsule	17848	41.9	14	7	7	10	7	0	9	7	0

Genomic islands known to contribute to bacterial fitness by conferring new properties increase the adaptability of the organism and may also encode genes involved in pathogenicity [52]. The analysis of the distribution of selected subtracted sequences and UPEC-associated pathogenicity islands (PAIs) amongst a panel of mucosa-associated *E. coli* isolated from colonoscopic biopsies of patients with colon cancer, patients with Crohn's disease and controls previously reported that neither the coloncancer nor the Crohn's disease mucosal *E. coli* populations are uniform [27]. In strain LF82, four PAIs were identified on the basis of homology with those characterized in ExPEC strains. However, some modifications were observed, such as the number of CDSs in the islands or their genomic organization. Differences were found when the presence of PAIs or the presences of genes encompassed in PAIs were compared in CD-associated *E. coli* strain LF82 and the commensal strain ED1a. PAI III, found in strain LF82, was absent in strain ED1a. Moreover, six additional CDSs in PAI I, two in PAI II and five in PAI IV were present in strain LF82. Interestingly, the insertion or deletion of genetic material events take place systematically at the same hotspots in LF82 genome than in various other *E. coli* genomes but different genetic information occurs at the same hotspot. These findings strongly suggest that further studies should be performed to investigate the role of these PAI-associated additional CDSs in strain LF82 virulence.

**Figure 5:**
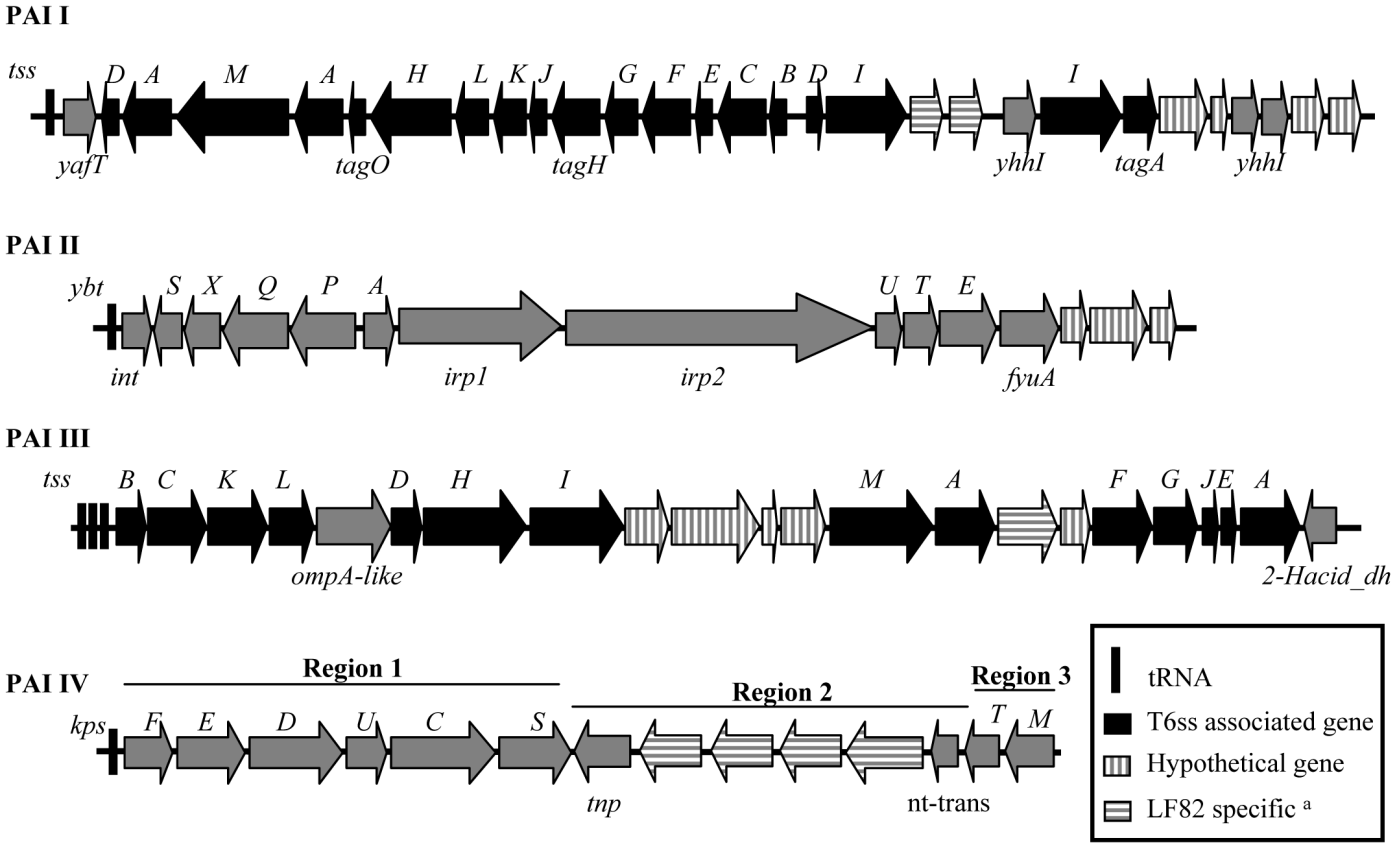
Genome organization of four putative pathogenic islands carrying virulence-related genes. PAI I and PAI III present genes encoding type VI secretion system (t6ss), PAI II is similar to the *Yersinia* high pathogenicity island and PAI IV present a similar genetic organization of group 2 capsule gene clusters. Black arrows indicate genes associated to t6ss, grey arrows indicated genes with assigned functions. Depending of the inclination, hatched arrows represents hypothetical proteins or proteins absents in any sequenced *E. coli* strains. Characteristic features of type VI secretion system genes products are indicated in Table S4.

The AIEC LF82 chromosome carries two putative type VI secretion systems located on PAI I and PAI III. This secretion system is a mechanism for Gram-negative bacteria to export proteins across the cell envelope [53], were identified in *Vibrio cholerae* [54], *Pseudomonas aeruginosa* [55], *Burkholderia pseudomallei* [56], *Burkholderia mallei* [57], *Edwardsiella tarda* [58], APEC [59], EAEC [60] and UPEC [50], [52], [61]. Such a presence of two different type VI secretion systems is found in B2 strains 536, UTI89, APEC-01 and S88. In contrast, strains CFT073 and ED1a possess only one type VI secretion system, located in PAI III and PAI I, respectively, and the EPEC strain E2348/69 does not possess any. Strain LF82, PAI I, inserted at the tRNA *AspV*, has a gene organization similar to that observed in most ExPEC strains except strain CFT073, with two *tssD* genes encoding Hcp-like proteins, a *tssH* gene encoding clpV-1 ATPase, a *tssI* gene encoding VgrG homologue and two distantly related *tssA* genes encoding ImpA homologues (Figure 5, Table S5). However, the LF82 PAI I harbors 29 CDSs as against 26 CDSs for UTI89, S88, 536 and APEC_01 or 23 for ED1a (Table 5, Figure 5). Among the additional genes found in LF82 PAI I, we identified the gene *yhhI* encoding a transposase and two specifcs CDSs encoding hypothetical proteins sharing strongest homologies with CSAG_00872 and CSAG_00871 conserved hypothetical proteins from *Citrobacter* spp. With the presence of the transposase encoding gene *yhhI*, we observed a duplication of the gene *tssI* encoding the VgrG protein. However, during the duplication event it is notable that one of the duplicated genes was truncated in the 3′ region, which eliminated the C-terminal extension corresponding to the effector domain of VgrG protein. PAI III is the other island that also encodes a type VI secretion system. The LF82 PAI III contains 22 CDSs, as in strain CFT073, as against 16 to 20 in most of the B2 strains. PAI III is absent in ED1a and E2348/69 strains. The PAI III of LF82 strain harbors classical genes involved in type VI secretion system encoding Hcp-like protein, clpV ATPase and VgrG homologue. It also harbors one specific CDS encoding hypothetical protein sharing highest homologies with ykris0001_25080, a hypothetical protein from *Yersinia kristensenii* ATCC 33638.

The LF82 PAI II, located close to the *AsnT* tRNA site, is similar to the core region of the “high pathogenicity island” (HPI) of pathogenic *Yersinia* sp. and encodes the yersiniabactin siderophore system [62], [63]. LF82 PAI II, also referred as PAI IV in UPEC strain 536 or PAI-*asnT* in strain CFT073, harbors 15 genes or CDSs like all the B2 strains, except strain ED1a, which has only 13 CDSs, and is absent in the EPEC strain E2348/69. LF82 PAI II encompassed *irp1* and *irp2* genes encoding non-ribosomal peptide synthetases polyketide synthases (NRPS_PKS) [64]. In strain LF82, these genes are probably functional since we did not observe an in-frame stop codon like in strain CFT073, which blocks yersiniabactin expression. Analysis of the core genome clearly showed that PAI II could be extended not only in strain LF82 but also in the other B2 strains (Table S2). Finally, we observed the presence of three additional CDSs encoding putative adhesins and invasins. The role of yersiniabactin biosynthesis in AIEC LF82 gut colonization should be investigated since FyuA, the outer membrane receptor for yersiniabactin [65], is one of the most highly up-regulated genes in biofilm formation in UTI strains [66] and because biofilm formation is a phenotypic feature of AIEC [21].

The LF82 PAI IV, located close to the *phe*V tRNA site, shares similarities with PAI V of strain 536 or PAI-*pheV* of strain CFT073. It contains gene clusters encoding for group 2 capsule involved in UPEC strain 536 in a murine model of ascending urinary tract infection [67]. The LF82 gene cluster encoding capsule has a similar genomic organization than that described for K1 and K5 capsule synthesis [68]. Regions 1 and 3, encoding for proteins involved in the secretion of capsule components, have the same genes in strain LF82 and K1 and K5 *E. coli* strains. Region 2, defined as a highly variable antigen-specific region, contains four LF82 specific CDSs flanked by CDSs encoding a transposase and a glycerol-3-phosphatecytidyltransferase.

### Identification of virulence genes in LF82 strain

We identified in strain LF82 virulence genes typically promoting motility, serum resistance, iron uptake, capsule and LPS expression, biofilm formation, adhesion to and invasion of epithelial cell lines. Of note, we found ten genes belonging to operons encoding known or putative fimbrial structures, including type1 pili and curli. Most of them are present in both pathogenic B2 strains and non pathogenic *E. coli* K-12, except the *auf* and *ygi* operons. Both are absent in strains EPEC E2348/69 and K-12 MG1655 and the *ygi* operon is also absent in UPEC strain 536. In contrast, the UTI specific fimbriae-encoding genes *pap*, *foc* or *sfa*, allowing UPEC to bind to and to invade host cells and tissues within the urinary tract [69], were absent in strain LF82. Strain LF82 also differs in several virulence-associated traits that may correlate with its pathogenic potential (Table 6). Among virulence genes, we identified in strain LF82 the *ibeRAT* genes organized as an operon also present in strains UTI89 and APEC-01. It was originally described in an *E. coli* K1 strain isolated from a patient with human newborn meningitis [70]. Gene *ibeA* encodes an invasin for which several host receptors have been identified, such as Ibe10R on bovine brain microvascular endothelial cells (BMEC) [71], and vimentin and PSF protein on human BMEC (HBMEC) [72]. It also participates in first stages of colibacillosis in chickens by mediating interaction of APEC strains with lung epithelial cells [73].

**Table 6:** Virulence factor encoding genes found in LF82 genome outside pathogenicity islands.

		B2								K12
Gene(s)	Description	LF82	536	UTI89	CFT073	APEC_01	E2348/69	ED1a	S88	MG1655
*fimH*	Type 1 fimH adhesin	**+**	**+**	**+**	**+**	**+**	**+**	**+**	**+**	**+**
*csgA*	Curli	**+**	**+**	**+**	**+**	**+**	**+**	**+**	**+**	**+**
*yadC*	Uncharacterized fimbrial-like protein	**+**	**+**	**+**	**+**	**+**	**+**	**+**	**+**	**+**
*ydeQ*	Uncharacterized fimbrial-like protein	**+**	**+**	**+**	**+**	**+**	**+**	**+**	**+**	**+**
*yehA*	Predicted Yeh fimbrial-like adhesin	**+**	**+**	**+**	**+**	**+**	**+**	**+**	**+**	**+**
*yfcP*	Hypothetical fimbrial-like protein	**+**	**+**	**+**	**+**	**+**	**+**	**+**	**+**	**+**
*ppdD*	Putative major pilin subunit	**+**	**+**	**+**	**+**	**+**	**+**	**+**	**+**	**+**
*auf*	Putative fimbrial-like protein	**+**	**+**	**+**	**+**	**+**	−	**+**	**+**	−
*ygi*	Yqi fimbriae	**+**	−	**+**	**+**	**+**	−	**+**	**+**	−
*lpfA*	fimbrial-like protein	**+**	−	−	−	−	−	**+**	−	−
*gipA*	Peyer's patch-specific factor	**+**	**+**	−	**+**	−	**+**	−	−	−
*ibeA*	Invasion protein IbeA	**+**	−	**+**	−	**+**	−	−	−	−
*pdu*	coenzyme B12-dependent 1,2-propanediol catabolism	**+**	−	−	−	−	**+**	−	−	−
*ratA*	RatA-like protein	**+**	**+**	**+**	**+**	**+**	−	**+**	**+**	−
*fepC*	Ferric enterobactin transport ATP-binding protein fepC	**+**	**+**	**+**	**+**	**+**	**+**	**+**	**+**	−
*chuA*	Outer membrane hemin receptor chuA	**+**	**+**	**+**	**+**	**+**	**+**	**+**	**+**	−
*fhuA*	iron compound receptor (ferrichrome iron receptor)	**+**	**+**	**+**	**+**	**+**	**+**	**+**	**+**	−
*sitA-D*	*Salmonella* iron/manganese transport	**+**	**+**	**+**	**+**	**+**	−	**+**	**+**	−
*iss*	Serum survival	**+**	**+**	**+**	**+**	**+**	−	−	**+**	−
*sepA*	Extracellular serine protease	**+**	**+**	**+**	**+**	−	−	**+**	**+**	−
*yfgL*	Outer membraneVesicle formation	**+**	**+**	**+**	**+**	**+**	**+**	**+**	**+**	**+**
*ompC*	Outer membrane porin protein C	**+**	**+**	**+**	**+**	**+**	**+**	**+**	**+**	**+**
*ompA*	Outer membrane porin protein A	**+**	**+**	**+**	**+**	**+**	**+**	**+**	**+**	**+**
*nlpI*	Lipoprotein NlpI	**+**	**+**	**+**	**+**	**+**	**+**	**+**	**+**	**+**

We also observed in strain LF82 the presence of the *pdu* gene cluster, that contains 22 CDSs, involved in coenzyme B12-dependent 1,2-propanediol catabolism, propanediol being previously reported to be a crucial carbon source for *Salmonella* to be able to grow in the large intestine and replication within macrophages [74], [75]. Such gene cluster is absent in all B2 strains except the EPEC strain E2348/69. The presence of *pdu* operon in AIEC strain LF82 is of high interest since by homology with *Salmonella*, it should allow LF82 bacteria to better colonize the intestine and to highly replicate within macrophages.

Another virulence gene identified in strain LF82 is *lpfA*, which belongs to the *lpf* operon encoding long polar fimbriae (LPF). Such an *lpf* operon is present in *Salmonella typhimurium*, *Shigella boydii* and *flexneri* and in enterohemorragic *E. coli* EDL933 [76], [77]. No role has been yet reported for LPF in *Shigella*. In *S.* Typhimurium, LPF promotes bacterial interaction with murine Peyer's patches (PP) [78]. For EHEC, experiments in pigs and sheep with O157:H7 strain 86-24 indicated that LPF contribute to intestinal colonization [79]. The presence of LPF encoding genes in strain LF82 could indicate that the AIEC bacteria are able to target Peyer's patches. Interestingly, we also identified in strain LF82 gene *gipA* that was first identified in *Salomonella Thyphimurium* [80] and whose expression is specifically induced in the small intestine. Gene *gip*A is also present in strains 536, CFT073 and in the enteropathogenic *E. coli* (EPEC) strain E2348/69. GipA allows *Salmonella* survival in PP and is involved in replication of intramacrophagic bacteria [74]. The presence of genes *lpfA* and *gipA* in strain LF82 is of great interest because clinical observations suggest that the sites of initial inflammation in ileal CD are the lymphoid follicles [81] and because microscopic erosions are observable at the specialized follicle-associated epithelium (FAE), which lines PP [82].

### Pathoadaptative mutations in AIEC strain LF82

**Figure 6:**
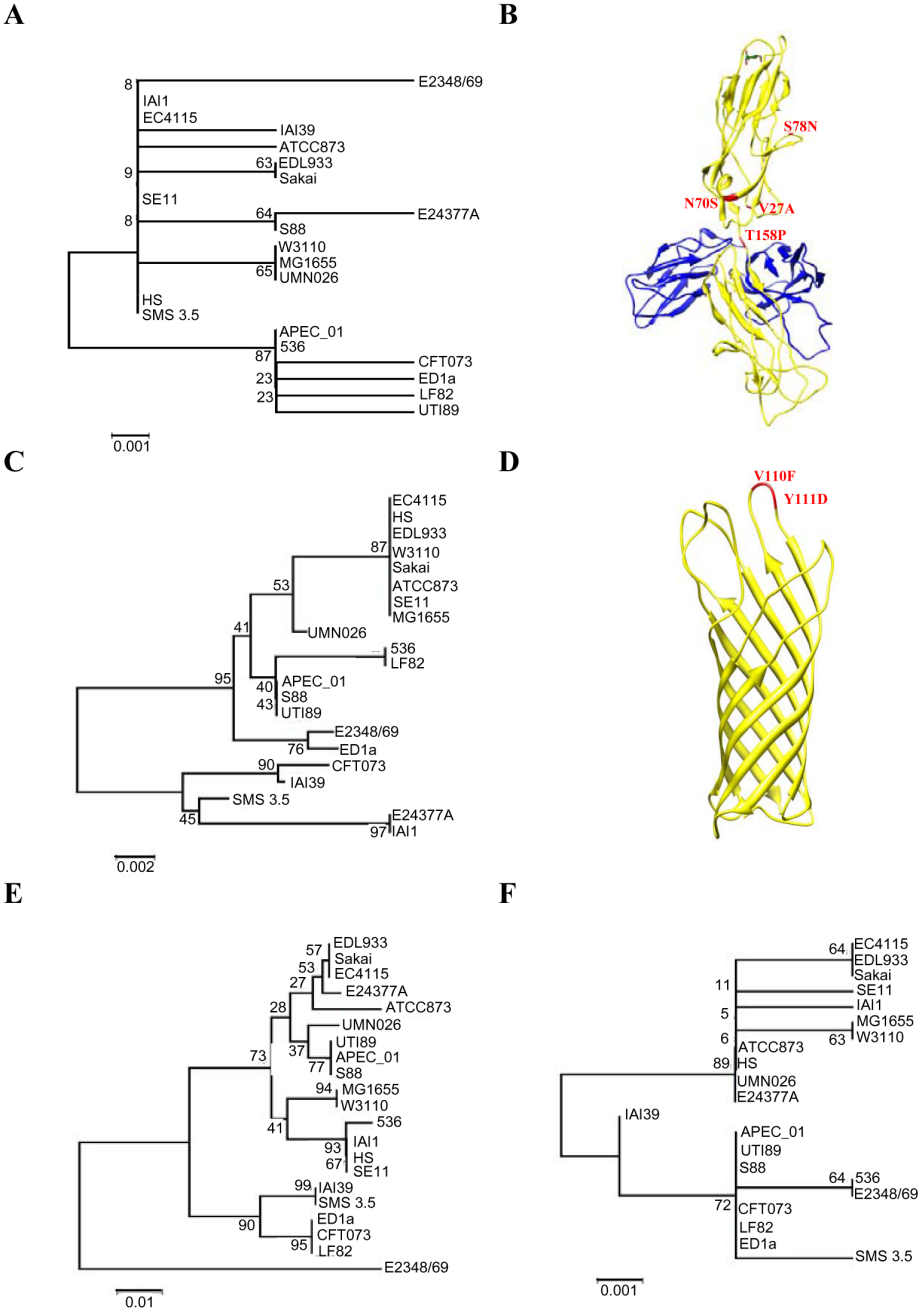
Phylogenic tree of sequenced *E. coli* strains. Phylogenic analysis were performed with FimH (A), OmpA (C), OmpC (E) and YfgL (F) variants. Location of substitutions in crystal structures are shown for FimH (B) and OmpA (D). FimH and OmpA are presented as ribbon in yellow [85], [95]. FimH structure is in complex with the chaperon FimC (blue ribbon) and mannose (stick). The substitutions are indicated in red. The percentage of replicate trees in which the associated taxa clustered together in the bootstrap test (500 replicates) are shown next to the branches.

We searched for pathoadaptative mutations in previously described LF82 virulence factor encoding genes such as *fimH* encoding the adhesin of type 1 pili, *ompA* and *ompC* encoding the outer membrane proteins OmpA and OmpC, and *yfgL* involved in outer membrane vesicle formation. The analysis of the amino acid sequences of FimH of all the *E. coli* strains so far sequenced indicated that the strains clustered in two major groups with one including all the B2 strains except the MNEC strain S88 and the EPEC strain 2348/69 (Figure 5A). Among the several substitutions in LF82 FimH, we found the N70S and S78N substitutions already described as specific to the B2 phylogroup [83], but also substitution T158P, which was not found in FimH sequences of all other *E. coli* strains so far sequenced. Interestingly, position 158 is located in the flexible loop which connects the pilin and the lectin domains (Figure 6B). The presence of such a substitution in strain LF82 is of great interest since it can induce a structural modification in this pilin lectin interdomain and amino acid substitutions in this interdomain, region were previously shown to increase the affinity of FimH for its ligand mannose [84].

The analysis of the OmpA amino acid sequences indicated that all B2 strains are located on a single major branch, except strain CFT073 (Figure 6C). The LF82 OmpA sequence is 100% homologous with that of strain 536. Both strains express a OmpA variant having V110F and Y111D substitutions located at the top of the inflexible external L3 loop [85], likely to be involved in the recognition of a host cell receptor (Figure 6D).

The analysis of OmpC amino acid sequence indicated that the B2 strains are clustered in two subgroups, one including the strains S88, UTI89 and APEC_01, and the second one with strains ED1a, CFT073 and LF82 (Figure 6E). In contrast, strain 536 was found in another cluster together with strains belonging to A and B1 phylogroups. The analysis of YfgL amino acid sequences indicated the presence of two major clusters with one including all the B2 *E. coli* strains (Figure 6F). The LF82 YfgL amino acid sequence is 100% homologous with to that of *E. coli* strains APEC_01, UTI89, S88, CFT073 and ED1a.

**Figure 7:**
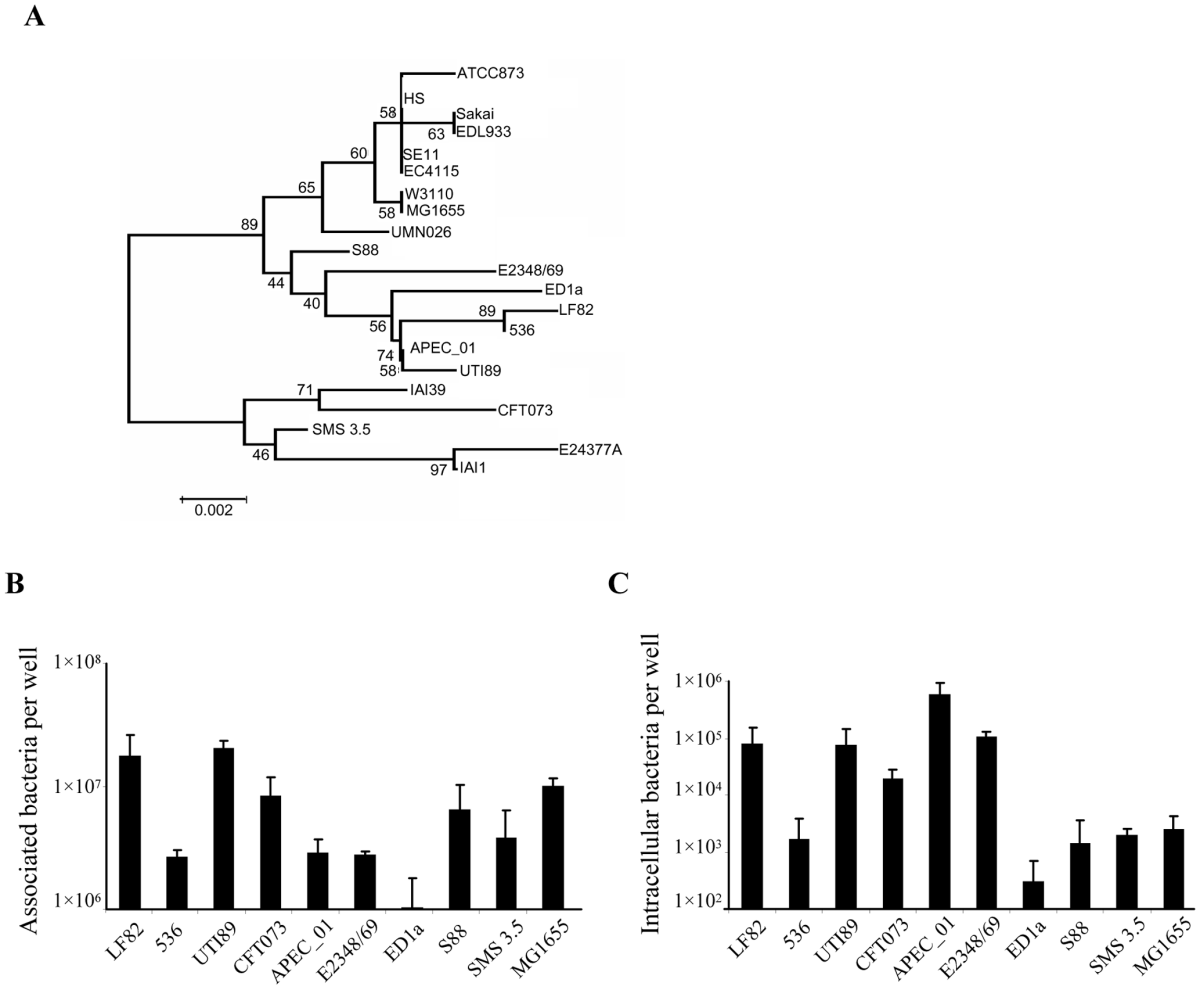
Phylogenic tree and studies on adhesion and invasion levels of *E. coli* strains. Location of LF82 in the phylogenic tree based on analysis of concatenated FimH and OmpA amino acid sequences (A), adhesion (B) and invasion (C) abilities of LF82 and other sequenced B2 or non pathogenic strains to Intestine-407 cells. The percentage of replicate trees in which the associated taxa clustered together in the bootstrap test (500 replicates) are shown next to the branches. Cell-associated bacteria were quantified after centrifugation and a 3-h infection period. Invasion was determined after gentamicin treatment for an additional 1h. Each value is the mean ± SEM of at least three separate experiments.

Regarding the role of these various virulence factors identified in LF82, we further analyzed the association of pathoadaptative mutations of two of them that play a major role in the interaction of the LF82 bacteria with host intestinal cells. FimH and OmpA interact with host receptors whose expression is abnormal in patients with Crohn's disease, ie the CEACAM6 glycoprotein acting as an intestinal receptor for FimH adhesion [23], [24] and the glycoprotein Gp96 involved in the fusion of *E. coli* outer membrane vesicle *via* OmpA to the plasma membrane of intestinal epithelial cells (Rolhion et al., in press). The analysis of the concatenated FimH and OmpA amino acid sequences indicated that the B2 strains divided into various clusters with one including strains LF82, 536, APEC_01 and UTI89 (Figure 7A). Interestingly, when we compared the adhesion and invasion levels of the *E. coli* strains belonging to this subgroup, we observed that strains LF82 and UTI89 adhered at a similar level and that like LF82, APEC_01 and UTI89 were highly invasive (Figure 7B and C).

### Conclusion

Strain LF82, belonging to phylogenetic group B2, is close to the avian pathogenic strain APEC_01, meningitis-associated strain S88 and urinary-isolated strain UTI89 on the basis of flexible genome and single nucleotide polymorphisms in various virulence factors. Comparison of the phenotype indicated the APEC-01 and UTI89 strains present similar ability to adhere to and to invade human intestinal epithelial cells, but LF82 differs from other B2 *E. coli* strains by its intermediate killer phenotype in mice. Based on these results it would be of high interest to compare the behaviour of APEC-01 and UTI89 strains to that of AIEC strain LF82 in an inflammatory bowel disease model, such as transgenic mice expressing the human CEACAM6 receptor [24] to investigate the potential of these strains to induce chronic inflammation in a compromised host.

We identified 115 AIEC LF82 CDSs (2.6%), not found in any *E. coli* genomes so far sequenced. In addition, 15 CDSs (0.3%) share no homology with genes so far identified in any pathogenic bacteria. Among known CDSs within the flexible genome, we found four pathogenicity islands, orphan genes, genes encoding many various virulence factors involved in adhesion, invasion, iron acquisition, serum resistance, proteases, propanediol catabolism, LFP and GipA important to target PP. Combined with host susceptibility factors, this could explain the pathogenicity of bacteria so far qualified as non pathogenic according to a modified Koch model taking into account the susceptibility of the host. It was recently reported that AIEC bacteria, among all the *E. coli* pathovars, were the only ones to benefit from autophagy deficiency, as observed in some Crohn's disease patients with mutations in IRGM and ATG16L1, and to better replicate intercellularly [86]. In addition, genome evolution in LF82 bacteria cannot be simply described by a “core genome and accessory gene pool” model. The analysis of the LF82 genome clearly showed that in addition to the presence of specific genes that could be involved in bacterial virulence, pathoadaptative mutations could also play a major role in making AIEC pathogenic in a compromised host. Analysis of single nucleotide mutations along the whole genome should be highly informative. For this purpose, genome sequencing of additional AIEC and non-AIEC strains should also be performed to better understand the AIEC-specific adaptations required to colonize the gut and to lead to the development of chronic inflammatory bowel disease in a genetic susceptible host.

## Materials and Methods

### Bacterial strain and Sequencing

*E coli* strain LF82 was isolated from a patient with Crohn's disease [10]. Reference strains used in this study are listed in Table 1.

For the LF82 genome project, a shotgun sequencing strategy using three different clone libraries and capillary Sanger sequencing was used to obtain a 12× coverage of the complete genome. For two of three libraries, genomic DNA was fragmented by mechanical shearing and 3 kb and 10kb inserts was respectively cloned onto pcdna2.1 (Invitrogen) and pCNS (pSU18 derived) plasmid vectors. In addition, a large insert (25 kb) BAC library was constructed from *Sau*3A partial digest and cloning onto pBeloBAC11. Vector DNAs were purified and end-sequenced using dye-terminator chemistry on ABI3730 sequencers. To reduce assembly problems due to repeated sequences, the assembly was realized using Phred/Phrap/Consed software package (www.phrap.com). The finishing step was achieved by primer walks, PCR and transposon bomb libraries and a total of 11984 sequences (670, 82 and 11232 respectively) were needed for gap closure and quality assessment.

### Genome annotation and Comparative genomic analysis

The LF82 chromosome and plasmid sequences were integrated into the MicroScope system [87] to perform annotation a comparative analysis with the other *E. coli* strains published in the context of the ColiScope project [48]. In addition, each protein of LF82 strain, manually validated, was compared with the genomes of *E. coli* strains 536, UTI89, CFT073, APEC_01, E2348/69, ED1a, S88, UMN026, AI39, SMS_3_5, EDL933, 157_H7, EC4115, E24377A, 55989, IAI1, SE11, ATCC 8739, HS, K12_DH10B, K12_MG1655 and W3110 using the TBLASTN program to also take into account potential unpredicted genes and genes with mispredicted start codons in the so far sequenced *E. coli* strains. A gene was considered conserved if the TBLASTN analysis produced an alignment with a minimum of 85% identity and over between 90 to 110% of the length of the query. In addition, TBLASTN analyses were manually validated to take into account a gene having a frameshift mutation. Finally, such genes were encompassed in the core genome.

To make easier the visualization of specific regions on the circular representation of the *E. coli* LF82 genome, we created color gradient that denotes the percentage of organisms which possess a homolog of a given gene of the reference genome. If this particular gene is present in all the organisms under study, it is tagged in light color (blue, red or green). Conversely, if it is only present in the reference genome, it is tagged in dark color (blue, red or green). In other words, the more pronounced the color, the higher the specificity.

Conserved gene clusters, i.e., synteny groups, were computed according to Vallenet *et al.* [88]. The Synteny plots has been obtained using the MaGe graphical interface of the ColiScope project (https://www.genoscope.cns.fr/agc/mage).

### Mage informatics Tool *RGPfinder*

Regions of Genomic plasticity (RGPs) of the LF82 genome were searched in the *E. coli* strains 536, UTI89, CFT073, APEC_01, E2348/69, ED1a, S88, UMN026, AI39, SMS_3_5, EDL933, 157_H7, EC4115, E24377A, 55989, IAI1, SE11, ATCC 8739, HS, K12_DH10B, K12_MG1655 and W3110 genomes with the web tool *RGPfinder*, implemented in the annotation platform MaGe (http://www.genoscope.cns.fr/agc/mage; Roche *et al.*, in preparation).

*RGPFinder* searches for synteny breaks between a reference genome and a set of closely related bacteria, named the Bacterial genome set. A region of genomic plasticity (RGP) *sensu lato* is the sum of overlapping sub-regions that are missing in at least one of the bacterial genome comparison set. RGPs have a minimal size of 5 kb. This definition does not make any assumption about the evolutionary origin or genetic basis of these variable chromosomal segments. *RGPFinder* also provides information about composition abnormalities (GC% deviation, Codon Adaptation Index) and RGPs flanking features such as tRNA, IS, integrase (int) and genetic elements involved in DNA mobility (mob) which are common characteristics of foreign DNA acquired by horizontal genetic transfer such as Genomic Islands (GI) and prophages (P).

### Phylogenetic analysis

The phylogentic analysis of FimH, OmpA, OmpC and YfgL was inferred using the Neighbor-Joining method [89]. Bootstraps were defined on 500 replicates. The evolutionary distances were computed using the Poisson correction method and are in the units of the number of amino acid substitutions per site. Horizontal branches are drawn proportional to inferred evolutionary distance. Phylogenetic analyses were conducted in MEGA4 [90].

### Multilocus Sequence Typing

The multilocus sequence typing (MLST) was performed as previously described by Jaureguy et al. [42]. This MLST scheme used internal portions of the eight housekeeping genes *dinB*, *icdA*, *pabB*, *polB*, *putP*, *trpA*, *trpB* and *uidA*. ClonalFrame [91] was used with 100,000 iterations, including 50,000 burn-ins to infer a recombination-insensitive phylogeny from the MLST data [90] to draw the consensus phylogenetic tree obtained using ClonalFrame.

### CRISPR identification

Identification of CRISPR loci in LF82 genome and searches for spacer homologs were performed as previously described [92].

### *In vitro* adhesion and invasion assays and *In vivo* virulence analysis

The bacterial adhesion and invasion assays were performed using the human intestine cell line Intestine-407 as previously described [93].

A mouse model of systemic infection was used to assess the intrinsic extraintestinal virulence of *E. coli* strains [94]. Mice were challenged subcutaneously with a standardized bacterial inoculum of 2×10^8^.and mortality was assessed over 6 days post-challenge. In this model system, lethality is a rather clear-cut parameter and, based on the number of mice killed, strains are classified as non-killer (<2 of 10 mice killed), killer (>8 mice killed) [49] or intermediate.

## Supporting Information

Table S1 LF82 plasmid composition compared to *Salmonella* and *Yersinia*.
(0.03 MB XLS)

Table S2 Auxiliary genes of LF82 chromosome genome compared to other complete *E. coli* genomes.
(0.55 MB XLS)

Table S3 Identification of phylogroup markers.
(0.04 MB XLS)

Table S4 Similarity search of the LF82 specific CDSs.
(0.03 MB XLS)

Table S5 PAI I and PAI III of LF82 strain: Standardized nomenclature for type VI secretion systems compared to other published nomenclature.
(0.03 MB XLS)
